# Microscopic Organisms as Instigators of Disease

**Published:** 1872-09

**Authors:** Ch. Raushenberg

**Affiliations:** Atlanta, Georgia


### MISCROSCOPIC ORGANISMS AS INSTIGATORS OF DISEASE.

BY CH. RAUSHENBERG, M.D., ATLANTA, GEORGIA.

The question of the origin and nature of certain very minute organisms which have frequently been observed to invest the solid and fluid constituents of the body in disease, and whether and why they must be considered the instigators of these diseases, has excited the interest of the medical profession for a number of years, and has become one of the most prominent questions of the present day.

An imperfect knowledge of the existence of very minute organisms has been entertained already for a considerable length of time, and has given rise to much discussion and controversy amongst biologists in relation to the question of their origin.

Spalanzani, in his "Opuscoli di Fisica Animale et Vegetale," Pavia, 1787, resorted to the so-called panspermatic hypothesis, which supposes that the atmosphere is filled with myriads of organic germs, in order to explain the origin of these seemingly self-generating organisms—an idea to which Redi, nearly a century before him, had already paved the way by his aphorism, "Omne vivum ex vivo," or "ex vovo," as it was afterward expressed. These conceptions were in full harmony with Spalanzani's and Stahl's theory of the existence of a so-called vital power, from which all the specific actions and faculties of organic bodies emanate, and which differs, in its manifestations and laws of action, from all the powers belonging to organic nature.

The older theory, of a spontaneous generation of living organisms (generatio acquivoca), was put to rest by these doctrines for some length of time, until it was revived again, and the contest renewed with increased vigor, by the French Academy of Sciences, about eight years ago, in an effort to account for the origin of these smallest known organisms.

The so-called panspermadists, with Pasteur as their leader, held that the development of ever so small and simple an organism is impossible, without the previous existence of a germ or egg from which to take its origin.

The heterogenists, with Pouchet* at their head, assert that the primary anatomical elements required for the existence of many minute and low plants and animals can be originated, under, certain circumstances, without the previous existence and coöperation of any living organisms.

It would not serve the more practical object of this paper to enter into the details of the animated controversy, as it was carried on by these parties. Numerous experiments and extensive investigations were made to ascertain whether life, in its lowest and minutest forms, could originate under the operation and influence of physical and chemical laws alone. Pasteur's efforts led to a negative result; those of Pouchet, and particularly of his followers, Dr. Childs and Dr. Ch. Bastian†, undoubtedly, to a very distinctly positive one. They discovered in glass tubes and retorts, filled with infusions of organic substances and air, after these fluids had been intensely boiled‡—after the air conducted there had passed through red-hot rotten stone, and after these tubes had been hermetically closed—more or less of these low organism, in all their different forms. Bastian, therefore, defends the possibility of a creation *de novo*, instead of *ex ovo*, which he calls *archebiosis*.

* G. Pennetier: L'Origin de la Vie. Paris, 1868. With a Preface, by Pouchet.

† The Modes of Origin of the Lowest Organism. London, 1871.

‡ Dr. Bastian submitted the contents of his glass retorts full four hours, to a heat of 140° to 150° Celsius.

Pasteur first established the fact, that many processes of fermentation depend, each one for itself, upon the presence and vital activity of certain specific organisms, and are instigated and maintained by them, and immediately interrupted by their death; while, if they continue, an infinite multiplication of the organism which produced them is the unavoidable consequence. The common yeast fungus, which is the instigator of the alcoholic fermentation of saccharine substances—their decomposition into alcohol and carbonic acid—furnishes the most common daily illustration of this process. Other and similar processes of fermentation are known to be instigated and developed by other fungi; for instance, the decomposition of a solution of tannin into gallic acid and sugar by penicillum glaucum and eurotium aspergillus niger.

With the hypothesis that the process by which infectious diseases originate from and multiply their specific contagion is analogous to a process of fermentation which gained for them the name of zymotic diseases, the supposition that living organisms may also originate these fermentations in the human system, as they otherwise do in nature, was simultaneously established; and with the origin of that idea, a careful search after living contagions and miasmata commenced.

These ideas were encouraged by the results of microscopic investigations, which revealed, beyond a doubt, that certain destructive epidemics of certain plants and insects are caused by the influence of certain organisms. The rot of the grape—that of the Irish potato, the muscadine and gattine—epidemic diseases of the silkworm, and one of the house fly, which has often been observed in the fall of the year—were subjected to minute investigation. The mode of infection, the connection between the progress of the disease, and the increased development of the fungi, were carefully observed, and confirmed by exact experiments as the results of the development, growth and multiplication of certain low organisms.

Many researches have since been made to discover similar organisms as the causes of the infectious diseases of the human system. Much material has accumulated, and many opinions have been advanced and adopted by different investigators; but no definite general results have yet been attained and substantiated with absolute certainty. The subject, however, is daily gaining more interest and importance, and deserves more and more the general attention of the profession.

It would require too much space to enter into a detailed description of the organization and vital action of these low organisms. They have been divided by botanists into two large groups; of which the larger one belongs to the lowest form of vegetable life, the family of the mould-fungi—the smaller one to the so-called schizomycetes—of which several forms exhibit such free, apparently voluntary motion, that Prof. Rindfleisch's remarks about their appearance under the miscroscope must appear strikingly correct to every one who has seen and observed these formations. He says: "Then we have the common image, where a many-linked chain, with the addition of a little water, or with a slight pressure on the glass cover, suddenly separates into different points. These points elongate a little, and represent the image of the common bacterium, which is known to every observer by its unusually versatile and social conduct. I can not make up my mind to take these creatures for plants. The character of their motion is that of voluntary action as animals alone possess it. They swim along, for instance, with unusual velocity in a straight line; finding an object in their way, they stop, swim round it in different directions, as if examining it, then move on again smaller and larger distances in different directions. From time to time they rest; then perhaps a whole colony crowds together, and approaching each other closer and closer, they form those apparently regularly-arranged gelatine discs, which F. Cohn has called zoogloca, etc*."

While, therefore, there exists no doubt as to the position which the larger group of these low organisms occupy—I mean that of the mould-fungi—the character of those just described is by no means so certain, and their classification amongst the plants is at least doubtful.

The schizomycetes are remarkably small—of round, oval or cylindrical form; appear frequently single, frequently combined

*Untersunhungen über Niedere Organismen, von Prof. Dr. Rindfleisch. *Virchow's Archiv.*, Vol. liv, page 399.

together in rows, or long, but always single, undivided filaments. The single cells show a single, not double, contoured cell membrane. They are mostly colorless—sometimes yellow, blue, green, red, etc., in consequence of the imbibition of the specific products of fermentation initiated by their presence. Sometimes they are without motion; but often, again, they exhibit more or less vivid motory action. Some shoot about very lively from place to place; some show an oscillatory, some a serpentine motion. They multiply by division. The propagation of their species becomes possible because, with the evaporation of the fluids in which they are contained, a large number of them are transferred to the atmosphere. Whenever they come again in contact with suitable fluids, they continue to increase by increased division, gaining their materials of growth from the products of the fermentation they have initiated.

The most of the organisms belonging to the family of the schizomycetes are bearers of certain substances, by which they establish peculiar processes of fermentation analogous to the alcoholic fermentation. They convert alcohol into acetic acid, milk sugar into lactic acid, and the more complicated processes of decomposition of the nitrogenous compounds (albumen, etc.) are initiated and maintained by them.

Bacterium termo is the common instigator of putrid decomposition. It appears in the form of very small, sharp-contoured round cells, of lively motion, as monas crepusculum; in that of staff-shaped, elongated cells of very different length, with oscillating motions, as bacterium, of which sometimes two or four united form a short chain; and in the form of longer filaments, consisting of a number of short cells, described by older observers as leptothrix, created by further division of the round and elongated cells just mentioned. These forms all belong to one species, and represent only different stages of development. Other forms of this group—for instance, the spirillum voluntas, thin, screw-shaped cylindric bodies, etc.—are not well known yet. Both groups together are often spoken of as plants of low organization, which can only grow on or within organic substances, as they are destitute of chlorophyl, and, therefore, of the faculty of the higher organized green-colored plants to develop organic material from air, water, and inorganic substances.

Amongst the parasitic fungi which produce local but less important changes on the human epidermis, the achorion schönleini, the trichophyton tonsurans, and the microsporon furfur, as the instigators of favus, herpes tonsurans (sycosis) and pityriasis versicolor deserve to be mentioned. The diseases caused by them are only local, but decidedly contagious. A severe inflammation of the subcutaneous cellular tissue, particularly of the lower extremities, followed by extensive inflammatory infiltration, purulent softening, sloughing and perforation of the tissues and skin above the fistulous cavities, occurs endemically in the East-Indies. It is known under the name madura-foot, mycetoma, and caused by a parasitic fungus, the chionyphe Carteri, which penetrates into the subcutaneous cellular tissue, and the soft parts underneath them, and causes such extensive and deep destruction, that severe surgical interference becomes frequently necessary to check its ravages.

Certain mould-fungi (aspergillus, penicillum, mucor, etc.,) which are occasionally found in the stagnant secretions of the ear, in ulcerating cavities of the lungs, etc., can not be considered specific instigators of disease. They only develop themselves on these organic substances, and promote their decomposition.

But these low organisms have not only been discovered on the surface of the human body, but often in internal organs, and in the blood in many diseased conditions of the human system. Often it appears plain that they are not the causes of the morbid process which they accompany, but merely the instigators of putrid decomposition in necrotic tissues and retained secretions; often, again, it is very difficult to decide which position they occupy to this process.

Leyden and Jaffé* discovered bacteria in their different forms in the sputis, and in the diseased parts in putrid bronchitis and gangrene of the lungs; and the writer has repeatedly had the opportunity to convince himself of the correctness of their ob-

* Leyden and Jaffé: Uber Putride Bronchitis. *Deutsches Archiv. für Klin. Medicin.* I., 489.

servation in relation to the occurrence of these organisms in the first-named disease. There is, however, no reason to consider them, in these cases, anything more than the instigators of putrid decomposition in the sloughing portions of the lung and the stagnating bronchial secretions.

Pyelo-nephritis, following chronic catarrh of the bladder, is often caused by bacteria introduced into that organ by catheterism. Traube* has furnished the proof for this fact, and has demonstrated that they convert, by their growth and multiplication, the simple catarrh of the bladder into a purulent one; that they find their way along the ureters into the pelvis of the kidney, and finally through the uriniferous ducts into the interstitial tissue of the kidneys, producing severe inflammation and suppuration, and more or less confluent abscesses, with an ample supply of bacteria.

The many observations and inquiries into the nature of the instigators of the different infectious diseases have yet led to very few positive results—partly on account of the great difficulty connected with the accurate and reliable observation of such organisms, their progress in the human system, and their effect on the different organs; partly on account of the failure of the investigators to prove by experiment that the peculiar disease in question could be artificially produced by introducing the suspected organisms into the bodies of healthy test animals. The zymotic hypothesis, however—or, in other words, the supposition that infectious and contagious diseases represent a process of blood-fermentation and decomposition initiated and maintained by the invasion, development and multiplication of minute vegetable or animal organisms in the human system—has at least been considerably strengthened and elevated from a mere apprehended possibility into a very reasonable probability, although we have only succeeded in proving these organisms to be the instigators of a few infectious diseases.

The results of Salisbury's investigations, according to which certain microscopic algæ were the carriers of the miasma and the producers of malarious fevers, have been proven to be unreliable.

* *Berliner Klin. Wochenschrift*, 1864, No. 2.

The choleraphyton of English, and later (1866), of German observers, and the cylindrotænium choleræ Asiaticæ—both suspected to be instigators of the disease—were recognized respectively as innocent ascaride eggs and as oidium lactis, a fungus of common occurrence on sour milk. Nevertheless, there is no disease whose course, mode of infection and propagation speak so much in favor of its origin by microscopic organisms as cholera.

Diphtheria is another disease which has been extensively investigated with an intention to prove its causation by microscopic organisms, and with a more satisfactory, but not finally conclusive, result.

Buhl (*Zeitschrift für Biologie* i, 341), Hüter (*Centralblatt* vi, 1868, 177), and Tomasi (*Centralblatt* vi, 1868, 531), observed monas crepusculum—the small, round, single-celled, quick-motioned bacterium form described above—in the diphtheritic coats of wounds and the patches of pharyngeal and laryngeal diphtheritis, and (the two last observers) also in the blood of such patients.

Oertel (*Bayerisch. Aerztt. Intelligenz Blatt.*, 1868, No. 31) has discovered the same organisms in the pseudo-membranes, the inflamed mucous surfaces, the lymphatics and lymphatic glands, the blood-vessels of the kidneys and other organs, and in the blood generally, in the proportion of six of them to one red corpuscle, and has frequently transferred the disease by inoculation. He considers them the contagium vivum of the local diphtheritic process from which the general infection of the system emanates.

These observations make the opinion of Oertel very probable; but as they do not contain any experimental proof that these organisms alone, and not the particles of diphtheritic membrane, or the fluids in which they are contained, are the bearers of the infection, this task yet remains to be performed by future observers.

Another disease which has been closely investigated for the presence of microscopic organisms, and their influence in originating it, is the murrain of the cattle. Although originally not a disease of the human system, its frequent transplantation to the same as pustula maligna imparts a good deal of medical interest to these investigations. Staff-like, motionless bodies were first discovered by Pollender, in 1855, and soon afterward by Brauell (*Virchow's Archiv.*, Vol. xi, 137, Vol. xiv, 432, Vol. xxxvi, 292) in the blood of living animals infected with this disease. Davaine (*Compt. Rend.*, lvii, 220, 351, 385—lix, 393, and *Memoires de la Societé de Biologie*, 1865, Ser. v., 393) had seen these organisms already in 1850, without ascribing any particular importance to them; but after Pasteur's researches on the influence of low organisms in the production of different processes of fermentation, he renewed his investigations, and convinced himself of the presence of these bodies, not only in the blood of the dead, but also of the living cattle infected with murrain; while he could never discover them in the blood of the healthy ones until about forty-eight hours after inoculation, when the first general symptoms of the disease made their appearance. Davaine takes these single staff-like bodies as a species of vegetable organisms different from, but related to bacterium termo, and considers himself justified, by the results of his inoculations, to pronounce them the instigators of the murrain. He found that the blood of inoculated animals would never produce the disease again if used for inoculation before these organisms (bacterides) had developed themselves within it, while it always did after they had made their appearance; and also that the blood from infested animals still contained these organisms well preserved after having remained in a dried state for months, and also retained its infecting power as long as it contained them.

The same observer has also proven the connection of pustula maligna with murrain by demonstrating that these organisms exist in the pustules and in the blood of persons diseased with them, and that the murrain can be produced in animals by inoculating them with the contents of the malignant pustule.

These discoveries of Davaine have been confirmed by later observations of Münch, Buhl, Waldeier and others, who noticed, besides the bacterides in the diseased parts and the blood, also small and large hemorrhagic infiltrations on the mucous membrane of the stomach and intestinal canal in human victims of this disease, which had been known a long time as a prominent pathological feature of it in animals.

Dr. Oscar Grimm, of St. Petersburg (Zur Pathologie des Milzbrandes—*Virchow's Archiv*, Vol. lix, page 262), denies, lately, the existence of microscopic fungi and bacteria in the blood of the living animals infected with murrain. He holds that they are formed only after the death of these animals—that they have nothing to do with the origin of the disease—and promises to give the detailed results of his investigations, and the reasons for his opinions, to the public before long.

Prof. Ferdinand Cohn, of Breslau (*Virchow's Archiv.*, Vol. lv, page 229), establishes, by late investigations, almost beyond a doubt, that certain very minute (1-300 to 1-500 of one line) so-called granular cells, already discovered by Keber, Zürn, Hallier (*Virchow's Archiv.*, Vols. xli and xlii), in the lymph of the vaccina and variola, are not fat globules, nor cell-centers, nor protoplasma-granules, nor granular deposits from the serum of the lymph, but *single-celled living organisms, belonging to the group of the ball-bacteria; and that they are not accidental admixtures from the atmosphere, but belong to the lymph of both diseases, already in the human system.*

The excessive caution which has been used in the execution of these experiments, and the importance of the results obtained, can only be appreciated by those who are acquainted, first, with the resources of microscopical technic to maintain an object designed for investigation pure and (if living and in course of development) unaffected by improper influences; and next, with the skill and reliability of the observer by whom they were made. He calls them mycrosphæra (ball-bacteria), and classes them with the family of the schyzomycetes and the group of bacteriaceæ, and considers it highly probable that they are the originators of a process of fermentation or chemical division of the lymph serum, the products of which, if they reach the circulation of an animal organism, cause a disturbance of normal life—a pathological process. In this light, he deems it probable that they are the producers of the contagion of the vaccina and variola, and is now engaged in testing the correctness of this hypothesis.

Amongst the few infectious diseases, however, in which low microscopic organisms have been discovered and scientifically demonstrated as the living virus producing the disease, pyæmia and septicæmia occupy the first rank. Kebs (*Beiträge Zur Pathol. Anatomie der Schusswunden*, Leipzig, 1872) established for himself an enviable reputation when he discovered the living virus of these pathological processes—its channels of entry and diffusion in the human organism, and its effects on the same. He found in the secretions of wounds, and in pus of all quality, more or less microscopic organisms—as minute round cells, either single and in live motion or attached to each other, forming lengthy filaments, or crowded together as thick quiet clusters, or elongated as staff-like bodies with an oscillating motion. They are evidently closely related to bacterium termo; but Klebs called them microsporon septicum—an unfortunate choice for a name, as it reminds the reader of the above-mentioned microsporon furfur, a fungus, and a very different formation. He discovered them as zoogloca on the granulating tissues and ulcerating cartilages of wounds, and established the fact that they produce inflammation and suppuration wherever they go, and thus enter the interstitial cavities of the conjunctive tissue, the marrow of the bones, producing traumatic osteo-myelitis, and even the blood vessels, by destroying their walls. There they produce thrombi at once, or are carried along until they settle at a distance from the wound at such localities where the blood-current is slower and less propulsive—for instance, behind the valves of the veins—and give rise to more or less irritation, sometimes to thrombosis, suppuration and softening of the thrombi, which always contain them in large numbers. If these thrombi are conveyed into some terminal artery, infarctus, with purulent softening and conical metastatic abscesses, are the consequences. If these organisms produce no thrombi at all, and only pass along with the blood-current into the capillary system of distant organs, they frequently give rise to inflammation and suppuration—for instance, in the liver—forming the well-known pyæmic metastic abscess.

He proved the correctness of his opinion by filtrating the secretions of wounds, containing large numbers of these animals, through earthern cylinders. The fluid thus divested of the organisms, when subcutaneously injected into animals, produced only more or less fever of a few days' duration—never local or metastatic suppuration, and never death; while the fluid containing them always produced extensive suppuration and death within a very few days.

As Klebs found these organisms alike in pyæmia and septicæmia, and was not able to establish a distinct difference between them anatomically, he consolidated them into one pathological process, and accounted for the difference of their course by the difference in the quantity of the organisms and the time in which they enter the body, and the amount of pyrogenous substances they develop within the blood.

This article was written with a desire to consolidate into a palatable form the most important general facts known, up to the present day, in relation to this subject. The main sources from which these facts given were principally obtained are: Uber Pflanzlische Organismen, von Dr. F. Steudner (*Volkman's Samlung Klinischer Vorträge*), and Der Ursprung des Lebens, von F. Hellwald (*Ausland*, No. 21, May, 1872.)

